# Does PGA external stenting reduce compliance mismatch in venous grafts?

**DOI:** 10.1186/1475-925X-6-12

**Published:** 2007-04-16

**Authors:** Zhong-zhao Teng, Guang-yu Ji, Hong-jun Chu, Zhi-Yong Li, Liang-jian Zou, Zhi-yun Xu, Sheng-dong Huang

**Affiliations:** 1 Aragon Institute of Engineering Research (I3A), University of Zaragoza, Spain; 2 Department of Cardiothocacic Surgery, Changhai Hospital, Shanghai, China; 3 Departments of Engineering & Radiology, University of Cambridge, Cambridge CB2 2QQ UK

## Abstract

**Background:**

Autogenous vein grafting is widely used in regular bypassing procedures. Due to its mismatch with the host artery in both mechanical property and geometry, the graft often over expands under high arterial blood pressure and forms a step-depth where eddy flow develops, thus causing restenosis, fibrous graft wall, *etc*. External stents, such as sheaths being used to cuff the graft, have been introduced to eliminate these mismatches and increase the patency. Although histological and immunochemical studies have shown some positive effects of the external stent, the mechanical mismatch under the protection of an external stent remains poorly analyzed.

**Methods:**

In this study, the jugular veins taken from hypercholesterolemic rabbits were transplanted into the carotid arteries, and non-woven polyglycolic acid (PGA) fabric was used to fabricate the external stents to study the effect of the biodegradable external stent. Eight weeks after the operation, the grafts were harvested to perform mechanical tests and histological examinations. An arc tangent function was suggested to describe the relationship between pressure and cross-sectional area to analyse the compliance of the graft.

**Results:**

The results from the mechanical tests indicated that grafts either with or without external stents displayed large compliance in the low-pressure range and were almost inextensible in the high-pressure range. This was very different from the behavior of the arteries or veins in vivo. The data from histological tests showed that, with external stents, collagen fibers were more compact, whilst those in the graft without protection were looser and thicker. No elastic fiber was found in either kind of grafts. Furthermore, grafts without protection were over-expanded which resulted in much bigger cross-sectional areas.

**Conclusion:**

The PGA external extent contributes little to the reduction of the mechanical mismatch between the graft and its host artery while remodeling develops. For the geometric mismatch, it reduces the cross-section area, therefore matching with the host artery much better. Although there are some positive effects, conclusively the PGA is not an ideal material for external stent.

## Background

Autogenous vein grafting is widely used in regular bypassing procedures in the treatment of ischemia due to occlusive vascular lesions, such as atherosclerosis. However its patency is limited by progressive intima hyperplasia, which causes serious clinical problems, needing repeated angioplasty and graft surgery. Extensive studies have been carried out for capturing the mechanisms of restenosis. Currently the mechanical property/compliance and geometric mismatches are regarded as the main factors contributing to graft failure. Due to the poorly developed venous middle layer, the graft is over-expanded in an arterial environment where the blood pressure is much higher than that in the vein. The over-expansion induces extremely high stresses in the venous wall, approximately 5 times those in the artery wall and 140 times those in the normal vein [1], which definitely damages the living components in the venous wall [2-4]. The distension also leads to a geometrical mismatch around the anastomosis, where the eddy flow forms [5-7] hence causing disordered shear stress distribution and long particle residence time.

Limited by these mismatches, vein patency is generally less good than those of selected arterial grafts, such as mammary artery [8], the right gastro-epiploic artery [9], and the radial artery [10], whose mechanical property meets that of their hosts much better. However, most arteries are necessary, so available arterial conduits are very limited. Therefore this generates an interest in looking for approaches to increase the patency of venous grafts. One of the most efficient is called external stenting (ES), which was first introduced by Parsonnet *et al*. [11] by using a sheath to cuff the vein section to reduce the mismatch between graft and host. It has been shown that this protection could preserve the endothelium cells (ECs), smooth muscle cells (SMCs) and elastic fibers and increase vasa vasorum [12,13]. It also indicates that the ES can decrease wall thickness and matrix deposition significantly [13-15], prevent atherosclerosis around the anastomosis [16-20] even in hypercholesterolemic animals [15], and reduce cell apoptosis [3,7,21,22]. Moreover, ES can not only improve the patency of the normal vein graft, but also means that varicose and dilated veins can be used [23,24].

Up to now, investigations on ES have been largely focused on morphological, histological and immunochemical aspects, whilst quantitative results of the mechanical properties of the graft with ES are still little known, particularly, whether or not the compliance mismatch can be eliminated, with the degrading of ES remaining unknown. In this study, the compliance and distensibility of venous grafts with and without ES were investigated. Studies were performed on hypercholesterolemic rabbits. The jugular vein was harvested and implanted into the carotid artery transpositionally to form an end-to-end graft and externally stented with a non-woven polyglycolic acid (PGA) fabric. After 8 weeks the animal was sacrificed. The graft was harvested for mechanical tests as well as morphological and histological examinations.

## Methods

### Animal model

Twenty-five adult male rabbits, with a weight ranging from 2.0 to 2.5 kg, were divided into 5 groups at random (5 per group). One group was given a normal diet, named as NDG, and the other four groups, named as HSM, HM, HSH and HH, respectively, were given a hypercholesterolemic diet (HD) by the additional of 1 *g *of cholesterin powder per day. The grafts from groups HSM and HM were used for mechanical study, and the grafts from groups HSH and HH were used for morphological and histological examinations (Please refer to the **Abbreviations **for details of each group). At the beginning of week 0,2,4,8 and12, blood samples were taken from all the rabbits from groups NDG and HH to perform total cholesterol (TC), triglyceride (TG), low density lipoprotein (LDL) and high density lipoprotein (HDL) examinations (BIO-RAD3550-UV, Baxter, USA). Rabbits were fasted 12 hours before blood extraction via ear edge vein. At the end of the 12^th ^week, the grafting operations were carried out on all hypercholesterolemic animals and external stents were used for groups HSM and HSH. The diet remained unchanged after the operation.

#### Main procedures of the grafting operation

**Figure 1:**
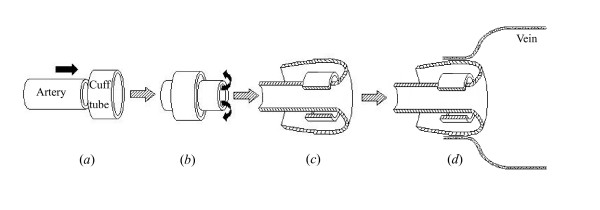
Schematic drawing of the grafting process (the silk thread is not shown here).

(1). The feeding was stopped 12 hours before the surgery and the water supply was stopped 4 hours before. The animal was anaesthetised with 1% nembutal solution (35–40 *mg*/*kg*) and heparinized with a dose of 1 *mg*/*kg*. An additional nembutal solution (5–10 *mg*/*kg*) dose was injected if needed during the operation.

(2). The jugular vein and carotid artery were dissociated totally by layered incisions. Then the jugular vein, around 3.5 *cm *in length, was cut and the residual blood was washed away carefully with saline solution containing narceine (1 *mg*/*ml*) and heparin (1 *mg*/*ml*). The blood flow in the artery was stopped by non-trauma serrefines, and a section, around 1.5 *cm *in length, was cut away perpendicular to the axial direction. A cuff technive [25,26] was adopted to make the implantation, which is shown in Figure 1. Briefly, pass the carotid artery through a 3 *mm *length cuff tube (see Figure 1 (*a*, *b*)); overturn the end of the artery to enwrap the cuff tube (see Figure 1 (*c*)) and tie with 3-0 silk thread; connect the jugular vein and the artery by another 3-0 silk thread tie (see Figure 1 (*d*)). The connection close to the heart was made first so that air could be expelled from the vein section and then the distal end was connected and tied to form an end-to-end graft. The cuff tube used here was obtained from polyethylene infusion tube with a 2.1 *mm *inner diameter and a 0.25 *mm *wall thickness, which was strengthened by being immersed in 90% alcohol solution for 48 hours.

(3). For the groups (HM & HH) without ES, the serrefine close to the heart and the one at the distal end were released to reopen the blood flow. The operating area was then closed in layers with 4# silk thread.

(4). For the groups (HSM & HSH) with ES, when the target vein was exposed, its outer diameter was measured at both ends and the middle point to calculate the width of the ES. The length of the ES had to be sufficent to cover both cuff tubes. Therefore a rectangular piece of PGA non-woven fabric (provided by GUNZE LTD, Japan) was made to wrap the graft and sutured with 6-0 proline line (Johnson & Johnson Medical LTD., USA). Then the operating area and wound were closed.

(5). A dose of 0.8-million units of penicillin was administered as soon as the operation was completed and 3 days later. Aspirin was administered at 12.5 *mg*/*kg*/day in the post-operative 4 weeks. The hyper-cholesterolemic diet was continued on the following day.

### Histological test

The animals in groups HSH and HH were used for histological examinations. 8 weeks after the operation, the graft, around 3 *cm *in length, was harvested. Snipping off 0.5 *cm-*long sections at both ends, the remaining part was cut into 4 rings and 3 were selected arbitrarily for morphological and histological examinations: the thickness of the intima and media was measured by the Leica Qwin image analysis system (U.K.); victoria blue-ponceaux (VB) was used to stain elastic fiber and collagen fiber.

### Mechanical test

The grafts and the joined arteries (0.5–1 *cm*) from the animals in groups HSM and HM were harvested and submerge in ringer solution containing narceine (1 *mg*/*ml*) immediately and preserved at 4°C for mechanical tests. The non-touched artery and vein (around 3.5 *cm*) opposite to the graft were also harvested (the arteries were grouped as HA, and the veins were HV). All vessels were mounted in an apparatus built in our laboratory (I3A, Spain) to perform expansion tests in 6 hours after cutting. A detailed description of the experimental apparatus has been well presented elsewhere [27,28]. The sensors used in the apparatus are the following: 1. LVDT (SX 20 ME × 200, Sensorex, France) for measuring the volume of the liquid pumped into the specimen; 2. Microliter syringe (Hamilton, Switzerland); 3. Pressure sensor (TP3, AEP, Italy) for measuring the pressure; 4. A/D card (NI PCI 6014, National Instrument, USA) for recording the digital signals.

It has to be pointed out that the graft with protection usually had many vasa vasorums, so some of the bigger ones were ligated carefully while those smaller ones were blocked by dried blood particles from the blood of the NDG group. The procedures of the expansion test are presented below.

#### Main procedures of the mechanical test

(1). The graft was cannulated at both ends until the tips touched the cuff tubes and then tied with 4# silk thread twice. The samples were submerged in sodium lactate ringer's injection (Guangdong Otsuka Pharmaceutical Co., Ltd.) and the temperature was controlled at 23°C by a dipped heater.

(2). Once mounted on the holders, the ringer solution containing blood particles was injected into the graft through a triple valve until the internal pressure could maintain at 50 *mmHg *in 1 minute without obvious decrease.

(3). The expansion test was performed by injecting the ringer solution with the microdose syringe. The amount of injected liquid was monitored by LVDT and the corresponding pressure was recorded continuously. The axial stretch ratio (*λ*_*z *_= *L/Lo *in which *L *is the stretched length and *Lo *is the length in the load-free state) varied from 1.0 to 1.4 and at each ratio the specimen was mechanically preconditioned three times. The testing procedure was repeated 5 times at each ratio and the loading stopped when the tube began to lose its straight profile. The pressure and volume (*p*-*V*) curve was therefore obtained. The maximum pressure (m*p*) in each *λ*_*z *_is listed in Table 1.

**Table 1:** Maximum pressure under different axial stretch ratios in different group (*mmHg*)

group m*p *(*mmHg*) *λ*_*z*_	1.0	1.1	1.2	1.3	1.4
HA	20	40	80	100	120
HV	10	10	20	30	40
HSM	20	30	80	160	160
HM	50	100	150	180	180

(4). After the mechanical test, the arteries were cut into rings (around 1 *mm *in length) and the geometric information was recorded to calculate the cross-section area (*Anon*-*load*) in load free state. The compliance of the system was measured by replacing the specimen with a rigid glass tube and it was taken into account in the experimental data analysis.

### Data fitting and statistics

In this study, the compliance rather than the stress-strain relation of the tissue was used to describe the mechanical property. This consideration has relevance to the compliance, giving the distensibility directly, which has already been well characterized clinically. The volume (*V*) data is converted into the equivalent cross-section area (*A*^*eq*^) by dividing it by the corresponding axial length (*L*). The relationship between pressure (*p*) and dimensionless change of *A*^*eq *^was used to describe the compliance of vessel and graft, which is defined as:

C=dΔA¯dp,
 MathType@MTEF@5@5@+=feaafiart1ev1aaatCvAUfKttLearuWrP9MDH5MBPbIqV92AaeXatLxBI9gBaebbnrfifHhDYfgasaacH8akY=wiFfYdH8Gipec8Eeeu0xXdbba9frFj0=OqFfea0dXdd9vqai=hGuQ8kuc9pgc9s8qqaq=dirpe0xb9q8qiLsFr0=vr0=vr0dc8meaabaqaciaacaGaaeqabaqabeGadaaakeaacqWGdbWqcqGH9aqpdaWcaaqaaiabdsgaKjabfs5aejqbdgeabzaaraaabaGaemizaqMaemiCaahaaiabcYcaSaaa@3645@

where ΔA¯
 MathType@MTEF@5@5@+=feaafiart1ev1aaatCvAUfKttLearuWrP9MDH5MBPbIqV92AaeXatLxBI9gBaebbnrfifHhDYfgasaacH8akY=wiFfYdH8Gipec8Eeeu0xXdbba9frFj0=OqFfea0dXdd9vqai=hGuQ8kuc9pgc9s8qqaq=dirpe0xb9q8qiLsFr0=vr0=vr0dc8meaabaqaciaacaGaaeqabaqabeGadaaakeaacuWGbbqqgaqeaaaa@2DCF@ is the dimensionless change of *A*^*eq*^, defined by

ΔA¯=Aeq−A0eqAnon−load,
 MathType@MTEF@5@5@+=feaafiart1ev1aaatCvAUfKttLearuWrP9MDH5MBPbIqV92AaeXatLxBI9gBaebbnrfifHhDYfgasaacH8akY=wiFfYdH8Gipec8Eeeu0xXdbba9frFj0=OqFfea0dXdd9vqai=hGuQ8kuc9pgc9s8qqaq=dirpe0xb9q8qiLsFr0=vr0=vr0dc8meaabaqaciaacaGaaeqabaqabeGadaaakeaacqqHuoarcuWGbbqqgaqeaiabg2da9maalaaabaGaemyqae0aaWbaaSqabeaacqWGLbqzcqWGXbqCaaGccqGHsislcqWGbbqqdaqhaaWcbaGaeGimaadabaGaemyzauMaemyCaehaaaGcbaGaemyqae0aaSbaaSqaaiabd6gaUjabd+gaVjabd6gaUjabgkHiTiabdYgaSjabd+gaVjabdggaHjabdsgaKbqabaaaaOGaeiilaWcaaa@46C9@

in which *A*_0_^*eq *^denotes the *A*^*eq *^at zero pressure. Different vessels display different shapes of *p*-ΔA¯
 MathType@MTEF@5@5@+=feaafiart1ev1aaatCvAUfKttLearuWrP9MDH5MBPbIqV92AaeXatLxBI9gBaebbnrfifHhDYfgasaacH8akY=wiFfYdH8Gipec8Eeeu0xXdbba9frFj0=OqFfea0dXdd9vqai=hGuQ8kuc9pgc9s8qqaq=dirpe0xb9q8qiLsFr0=vr0=vr0dc8meaabaqaciaacaGaaeqabaqabeGadaaakeaacuWGbbqqgaqeaaaa@2DCF@ curve. For instance, artery displays an 'S' shape and that of the graft is convex in the entire pressure range. An arc tangent function can capture both of these shapes very well. Therefore it was introduced to fit the experiment data. This approach was used in a previous study[29].

ΔA¯
 MathType@MTEF@5@5@+=feaafiart1ev1aaatCvAUfKttLearuWrP9MDH5MBPbIqV92AaeXatLxBI9gBaebbnrfifHhDYfgasaacH8akY=wiFfYdH8Gipec8Eeeu0xXdbba9frFj0=OqFfea0dXdd9vqai=hGuQ8kuc9pgc9s8qqaq=dirpe0xb9q8qiLsFr0=vr0=vr0dc8meaabaqaciaacaGaaeqabaqabeGadaaakeaacuWGbbqqgaqeaaaa@2DCF@ = *a*·arctan (*b*·*p *+ *c*) + *d*

where *a*, *b*, *c *and *d *are material constants. Substituting ΔA¯
 MathType@MTEF@5@5@+=feaafiart1ev1aaatCvAUfKttLearuWrP9MDH5MBPbIqV92AaeXatLxBI9gBaebbnrfifHhDYfgasaacH8akY=wiFfYdH8Gipec8Eeeu0xXdbba9frFj0=OqFfea0dXdd9vqai=hGuQ8kuc9pgc9s8qqaq=dirpe0xb9q8qiLsFr0=vr0=vr0dc8meaabaqaciaacaGaaeqabaqabeGadaaakeaacuWGbbqqgaqeaaaa@2DCF@ in (3), the compliance (1) results in

C=a⋅b1+(a⋅p+c)2.
 MathType@MTEF@5@5@+=feaafiart1ev1aaatCvAUfKttLearuWrP9MDH5MBPbIqV92AaeXatLxBI9gBaebbnrfifHhDYfgasaacH8akY=wiFfYdH8Gipec8Eeeu0xXdbba9frFj0=OqFfea0dXdd9vqai=hGuQ8kuc9pgc9s8qqaq=dirpe0xb9q8qiLsFr0=vr0=vr0dc8meaabaqaciaacaGaaeqabaqabeGadaaakeaacqWGdbWqcqGH9aqpdaWcaaqaaiabdggaHjabgwSixlabdkgaIbqaaiabigdaXiabgUcaRmaabmaabaGaemyyaeMaeyyXICTaemiCaaNaey4kaSIaem4yamgacaGLOaGaayzkaaWaaWbaaSqabeaacqaIYaGmaaaaaOGaeiOla4caaa@404A@

An expansion rate is defined in this study to measure the expansibility of the vessel in a certain pressure range,

ηp1−p2=|Ap1eq−Ap2eqA120eq−A0eq|×100%,
 MathType@MTEF@5@5@+=feaafiart1ev1aaatCvAUfKttLearuWrP9MDH5MBPbIqV92AaeXatLxBI9gBaebbnrfifHhDYfgasaacH8akY=wiFfYdH8Gipec8Eeeu0xXdbba9frFj0=OqFfea0dXdd9vqai=hGuQ8kuc9pgc9s8qqaq=dirpe0xb9q8qiLsFr0=vr0=vr0dc8meaabaqaciaacaGaaeqabaqabeGadaaakeaaiiGacqWF3oaAdaWgaaWcbaGaemiCaa3aaSbaaWqaaiabigdaXaqabaWccqGHsislcqWGWbaCdaWgaaadbaGaeGOmaidabeaaaSqabaGccqGH9aqpdaabdaqaamaalaaabaGaemyqae0aa0baaSqaaiabdchaWnaaBaaameaacqaIXaqmaeqaaaWcbaGaemyzauMaemyCaehaaOGaeyOeI0Iaemyqae0aa0baaSqaaiabdchaWnaaBaaameaacqaIYaGmaeqaaaWcbaGaemyzauMaemyCaehaaaGcbaGaemyqae0aa0baaSqaaiabigdaXiabikdaYiabicdaWaqaaiabdwgaLjabdghaXbaakiabgkHiTiabdgeabnaaDaaaleaacqaIWaamaeaacqWGLbqzcqWGXbqCaaaaaaGccaGLhWUaayjcSdGaey41aqRaeGymaeJaeGimaaJaeGimaaJaeiyjauIaeiilaWcaaa@5A2F@

in which *A*_*p*_^*eq *^denotes the value of *A*^*eq *^when pressure equals *p*. From (Eq.(5)), ηp1−p2
 MathType@MTEF@5@5@+=feaafiart1ev1aaatCvAUfKttLearuWrP9MDH5MBPbIqV92AaeXatLxBI9gBaebbnrfifHhDYfgasaacH8akY=wiFfYdH8Gipec8Eeeu0xXdbba9frFj0=OqFfea0dXdd9vqai=hGuQ8kuc9pgc9s8qqaq=dirpe0xb9q8qiLsFr0=vr0=vr0dc8meaabaqaciaacaGaaeqabaqabeGadaaakeaaiiGacqWF3oaAdaWgaaWcbaGaemiCaa3aaSbaaWqaaiabigdaXaqabaWccqGHsislcqWGWbaCdaWgaaadbaGaeGOmaidabeaaaSqabaaaaa@349C@ reflects the relative expansion rate when pressure changes from *p*_1 _to *p*_2_.

The data in this study are presented as Mean ± SD. The comparison between two groups was tested using a Student-T test. p > 0.05 was used to indicate there is no statistical difference; 0.01 < p < 0.05 denotes significant statistical difference (marked with * or #) and p < 0.01 means a highly significant statistical difference (marked with ** or ##).

## Results

### Index of blood-fat

The index of blood-fat (TC, TG, HDL and LDL) of the rabbits in groups NDG and HH is shown in Table 2. It is clear that after 4 weeks of HD feeding, the level of blood fat increases significantly; after 8 weeks, the TC and HDL could be as much as 10 times higher than those in NDG. Thus in this study the hypercholesterolemic animal model is validated.

**Table 2:** The blood-fat level (*mmol/L*) (in each group n = 5)

**Index**	**Group**	**0-week**	**2-week**	**4-week**	**8-week**	**12-week**
**TC**	NDG	1.67 ± 0.43	1.72 ± 0.45	1.72 ± 0.25	1.98 ± 0.55	2.02 ± 0.58
	HH	1.80 ± 0.20	1.88 ± 0.22	2.59 ± 0.76*^#^	18.57 ± 1.25**^##^	19.08 ± 1.31**^##^
**TG**	NDG	1.06 ± 0.31	1.09 ± 0.45	1.11 ± 0.38	1.28 ± 0.35	1.36 ± 0.55
	HH	1.09 ± 0.15	1.12 ± 0.11	2.09 ± 0.87*^#^	3.83 ± 1.75**^##^	4.07 ± 1.54**^##^
**HDL**	NDG	0.61 ± 0.20	0.69 ± 0.26	0.65 ± 0.31	0.58 ± 0.17	0.76 ± 0.27
	HH	0.53 ± 0.07	0.55 ± 0.09	1.21 ± 0.35*^#^	2.04 ± 1.11**^##^	2.12 ± 1.23**^##^
**LDL**	NDG	0.93 ± 0.21	0.95 ± 0.33	1.01 ± 0.49	0.89 ± 0.27	0.98 ± 0.25
	HH	0.94 ± 0.26	1.06 ± 0.24	1.84 ± 0.36*^#^	14.68 ± 1.42**^##^	15.13 ± 1.64**^##^

Asterisk denotes the comparison with 0-week (*: 0.01 < P < 0.05; **: P < 0.01); Derrick denotes the comparison with NDG at the same temporal point (#: 0.01 < P < 0.05; ##: P < 0.01).

### Expansion rate and compliance

**Figure 2:**
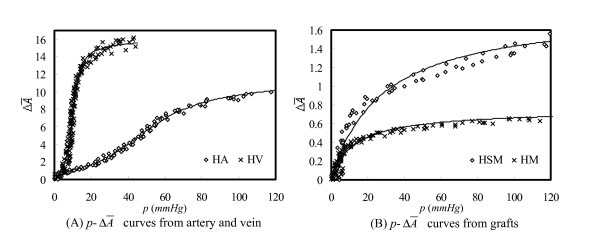
Experimental data points and regressed results (*λ*_*z*_= 1.4, data from HA1, HV1, HSM1 and HM1).

The arc tangent function (3) can fit the *p*-ΔA¯
 MathType@MTEF@5@5@+=feaafiart1ev1aaatCvAUfKttLearuWrP9MDH5MBPbIqV92AaeXatLxBI9gBaebbnrfifHhDYfgasaacH8akY=wiFfYdH8Gipec8Eeeu0xXdbba9frFj0=OqFfea0dXdd9vqai=hGuQ8kuc9pgc9s8qqaq=dirpe0xb9q8qiLsFr0=vr0=vr0dc8meaabaqaciaacaGaaeqabaqabeGadaaakeaacuWGbbqqgaqeaaaa@2DCF@ data for any kind of graft with any value of *λ*_*z*_. Representatively, the data from animals of HA1, HV1, HSM1 and HM1 (the first animal sacrificed in each group) at *λ*_*z *_= 1.4, are plotted in Figure 2 with the corresponding regression lines. The *p*-ΔA¯
 MathType@MTEF@5@5@+=feaafiart1ev1aaatCvAUfKttLearuWrP9MDH5MBPbIqV92AaeXatLxBI9gBaebbnrfifHhDYfgasaacH8akY=wiFfYdH8Gipec8Eeeu0xXdbba9frFj0=OqFfea0dXdd9vqai=hGuQ8kuc9pgc9s8qqaq=dirpe0xb9q8qiLsFr0=vr0=vr0dc8meaabaqaciaacaGaaeqabaqabeGadaaakeaacuWGbbqqgaqeaaaa@2DCF@ curves of non-touched arteries and veins display a representative 'S' shape (Figure 2A), especially the one from artery, while those from grafts are convex in the entire pressure range (Figure 2B). This difference may be due to the vein remodeling in the arterial environment.

Figure 2 indicates that the expansibility of the graft is much smaller than that of the vein. When pressure increases from 0 to 20 *mmHg*, *A*^*eq *^of the vein could increase more than 10 times (Figure 2A); however, that of the grafts only increased by about 50–80% (Figure 2B). Although the expansibility of the graft with ES was better than the one without protection, it was still much worse than that of the artery. Figure 2 also shows that for both vein and graft without ES, ΔA¯
 MathType@MTEF@5@5@+=feaafiart1ev1aaatCvAUfKttLearuWrP9MDH5MBPbIqV92AaeXatLxBI9gBaebbnrfifHhDYfgasaacH8akY=wiFfYdH8Gipec8Eeeu0xXdbba9frFj0=OqFfea0dXdd9vqai=hGuQ8kuc9pgc9s8qqaq=dirpe0xb9q8qiLsFr0=vr0=vr0dc8meaabaqaciaacaGaaeqabaqabeGadaaakeaacuWGbbqqgaqeaaaa@2DCF@ increases only a little when the pressure is more than 40 *mmHg*.

The quantitative comparison of expansibility is listed in Table 3. When pressure increased from 0 to 20 *mmHg*, *A*^*eq *^of the artery increased by about 20%, and was much less than those of the grafts, which were around 55% and 80%, respectively. This indicated that the wall of the grafts was quite loose. The expansion of the grafts without ES mainly appeared in the low pressure range, while when the pressure varied from 80 to 120 *mmHg*, *A*^*eq *^only changed 0.5 percent. For the graft with ES, *η*_80–120 _is about 3%, much lower than that of the artery (around 17%). Therefore, we can conclude from Table 3 that, in the physiological pressure range (80 to 120 *mmHg*), grafts with and without ESs are quite stiff.

**Table 3:** The expansion rate in different pressure ranges (*λ*_*z *_= 1.4)

	**HA**	**HSM**	**HM**
*η*_0–20 _(%)	20.20 ± 8.93	56.40 ± 7.34	82.43 ± 3.73
*η*_80–120 _(%)	16.55 ± 3.86	2.99 ± 0.92	0.45 ± 0.10

There is significant statistical difference between any two groups (P < 0.01).

**Figure 3:**
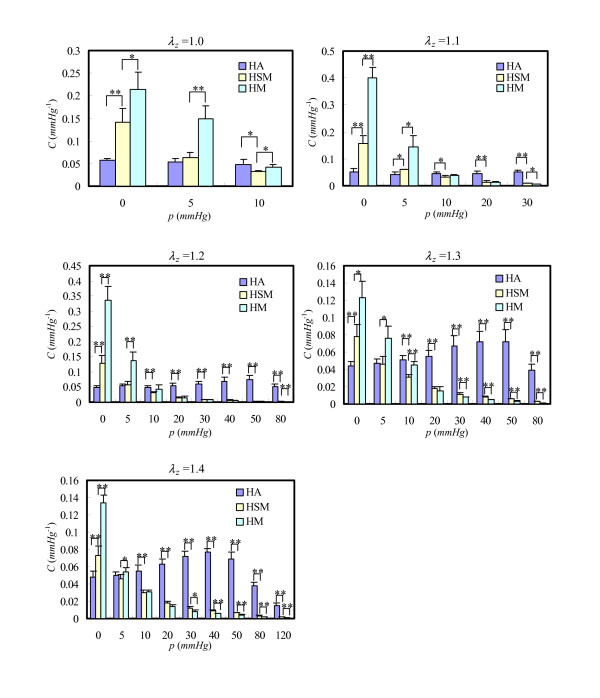
Comparison of compliance between grafts with external stent (HSM), without external stent (HM) and artery (HA). (* denotes 0.01 < P < 0.05; ** for P < 0.01).

**Figure 4:**
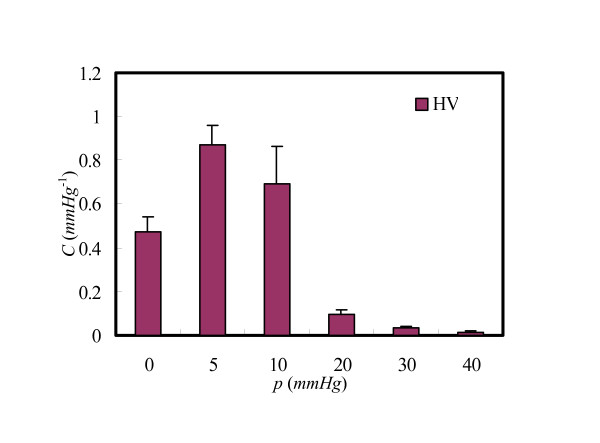
Variation of the venous compliance (HV) with pressure (*λ*_*z*_=1.4).

Since the artery, vein and graft are nonlinear materials, their compliances depend on the extra loading. From the definition (Eq.(4)), the compliance varies with *λ*_*z *_and the transmural pressure. Thus in order to have a complete analysis, the compliances at different *λ*_*z *_and pressure conditions are shown in Figure 3. Generally, for grafts, at any given *λ*_*z*_, their compliances decreased while *p *increased (see Figure 3); and with a given *p*, when *λ*_*z *_increased, it increased initially and then decreased. Due to the 'S' shape of *p*-ΔA¯
 MathType@MTEF@5@5@+=feaafiart1ev1aaatCvAUfKttLearuWrP9MDH5MBPbIqV92AaeXatLxBI9gBaebbnrfifHhDYfgasaacH8akY=wiFfYdH8Gipec8Eeeu0xXdbba9frFj0=OqFfea0dXdd9vqai=hGuQ8kuc9pgc9s8qqaq=dirpe0xb9q8qiLsFr0=vr0=vr0dc8meaabaqaciaacaGaaeqabaqabeGadaaakeaacuWGbbqqgaqeaaaa@2DCF@ curve of artery and vein, their shapes of compliance were convex in the pressure range, and for the artery, the peak point located around 45 *mmHg *(see graphs in Figure 3 with *λ*_*z *_= 1.2, 1.3 and 1.4), while that of the vein appeared around *p *= 5 *mmHg *(see Figure 4).

According to the conclusion from Table 3 and the values in Figure 3, the compliance of the grafts in the low pressure range (*p *< 20 *mmHg*) is much higher than that in the high pressure range (*p *> 20 *mmHg*). With a given *λ*_*z*_, when the pressure is less than 20 *mmHg*, the compliance of HM (without ES) was higher than that of HSM (with ES). Particularly, in the cases of *p *ranging from 0 to 5 *mmHg*, significant differences were found between HM and HSM. However, the decrease of compliance of HM with pressure was much faster than that of HSM. Therefore, when pressure exceeded 30 *mmHg*, the compliance of HSM was higher than that of HM, especially when *λ*_*z *_equaled 1.3 and 1.4.

Under the same pressure, the compliance of the vein was usually 10 times that of the graft (HSM or HM). This implies that the venous section becomes much stiffer in order to tolerate much higher loading in the arterial environment. With the protection of the ES, the mechanical property of HSM was more similar to that of the artery when this was compared with HM, even though there were still important differences in compliance (see Figure 3) and expansibility (see Table 3). Therefore at least 8 weeks after the grafting operation, the PGA ES does not have a significant impact on improving venous mechanical tone to make it similar to its host artery.

### Histological test

**Figure 5:**
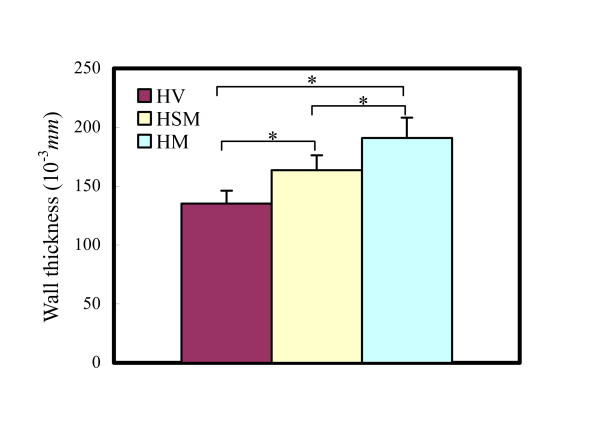
Thickness of intima and middle layer of HV, HSM and HM (* denotes 0.01 < P < 0.05).

The change of mechanical behavior due to remodeling should have a direct relationship with the change of micro components and structure, which could be examined by morphological and histological examinations. Numerous reports indicate that thickening happens during the remodeling process in grafts both with and without protection [13,15]. This study showed consistent results (see Figure 5); the wall of the grafts was much thicker than that of the vein (there was a significant statistical difference between HV and HSM/HM). The thickness of HSM was smaller than that of HM (there was a significant difference between them). The adventitial thickness of the graft is excluded in Figure 5 due to the blurred boundary caused by serious hyperplasy.

In fact, thickening is not detrimental to the graft, which decreases the high stress induced by the arterial blood pressure. However, ideal remodeling would be able to change the microstructure of the wall and finally arterize the venous graft. Now it is clear that remodeling leads grafts without protection to having a fibrotic wall, filled with foam cells, inflammation cells and, more likely to fail. The purpose of biodegradable ESs is to make remodeling develop well and finally eliminate the geometrical and mechanical mismatches between the vein and the host artery. However, the results in this study indicate that PGA ES does not guarantee an ideal remodeling process.

**Figure 6:**
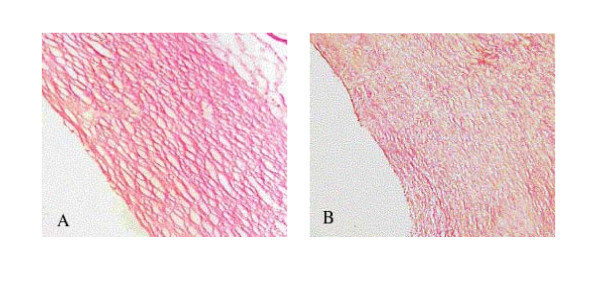
Collagen fiber in the graft wall (A: without external stent; B: with external stent; Stained with VB, × 200).

Apart from the thickness of the venous wall, the amount of elastic fiber and collagen fiber is another important factor in determining the mechanical property of the vessel wall. The collagen bundle in HM (without ES) is much bigger than the one in HSM (with ES), which is shown in Figure 6, but collagen fiber in HSM is more compact whilst that in HM is more floppy. Surprisingly we did not find any elastic fiber, either in HM or in HSM, which was possibly due to the HD. We found after 8 weeks of HD feeding that, the elastic fiber in the wall was getting significantly less and after 12 weeks it almost disappeared (data and picture are not shown in this paper). However, if normal vein is used, breakdown elastic fiber could be observed in the graft without protection, and the ES would preserve it [13]. The observation in this study means that the PGA ES does not promote elastin synthesis in the arterial environment.

The disappearance of elastic fiber may be the reason for the convex shapes of *p*-ΔA¯
 MathType@MTEF@5@5@+=feaafiart1ev1aaatCvAUfKttLearuWrP9MDH5MBPbIqV92AaeXatLxBI9gBaebbnrfifHhDYfgasaacH8akY=wiFfYdH8Gipec8Eeeu0xXdbba9frFj0=OqFfea0dXdd9vqai=hGuQ8kuc9pgc9s8qqaq=dirpe0xb9q8qiLsFr0=vr0=vr0dc8meaabaqaciaacaGaaeqabaqabeGadaaakeaacuWGbbqqgaqeaaaa@2DCF@ curve of the grafts (Figure 2(B)), the high expansibility (Table 3) and the high compliance in the low pressure range (Figure 3). It is well known that in the low pressure range, the load is mainly undertaken by elastic fiber, and when pressure exceeds a certain value, (for artery it is around 50 *mmHg*,) collagen fibers begin to tighten to provide strength for the wall, which is the reason of the 'S' shape of the arterial *p*-ΔA¯
 MathType@MTEF@5@5@+=feaafiart1ev1aaatCvAUfKttLearuWrP9MDH5MBPbIqV92AaeXatLxBI9gBaebbnrfifHhDYfgasaacH8akY=wiFfYdH8Gipec8Eeeu0xXdbba9frFj0=OqFfea0dXdd9vqai=hGuQ8kuc9pgc9s8qqaq=dirpe0xb9q8qiLsFr0=vr0=vr0dc8meaabaqaciaacaGaaeqabaqabeGadaaakeaacuWGbbqqgaqeaaaa@2DCF@ curve (Figure 2(A)). Grafts in this study lacking elastic fiber could be compared with a tuck net. During the early period of liquid being pumped in, pressure increases little, but as long as the volume reaches a certain value, tighting the collagen fibers, the pressure increases quickly. Thus the explanation for the mechanical behaviour of the grafts is the collagen fiberising of the wall.

## Discussion

From the foregoing characterization of the compliance and the micro-component of the wall, we have come to the conclusion that the PGA ES can improve the venous graft tone, giving rise to a smaller expansion rate in the low pressure range and a higher value in the physiological range (Table 3), a compliance closer to the artery and more compact collagen in the wall (Figures 6). However, the compliance of the venous graft with ES is still quite different from that of the artery. At least 8 weeks after the operation, the biodegradable PGA ES could not eliminate the mechanical mismatch between the venous graft and its host artery. Although we could not extend the validities of this conclusion for a longer period, (because after 8 weeks the PGA ES had not degraded thoroughly and the residual part could still be seen), we do not expect the improvement of graft tone to become similar to the artery after a longer period of remodeling.

The weakening of ES will allow the venous wall to undertake more and more loading and this causes tissue remodeling. Therefore the degrading property of ES should be important. According to technological reports from the provider, the PGA non-woven fabric used in our experiments degrades totally in 15 weeks in the saline solution *in vitro*. Actually, we found that after 12 weeks there was no perceptible remnant *in vivo *(the data is not shown in this paper). Despite its importance, the mechanical property evolution of ES *in vivo *is extremely poorly understood. Except for the weakening process, the degradation product is another important factor. The products of PGA are CO_2 _and H_2_O, which could decrease the local pH and affect the local tissue, possibly promoting apoptosis.

The PGA we used is quite rigid. The Young's module is 11.371 *Mpa*, which means it can be regarded as an inextensible material in the physiological pressure range, since the strain is less than 0.5 × 10^-3 ^under 100 *mmHg *transmural pressure. Therefore the PGA ES could not eliminate mechanical property mismatch initially. Zweep *et al*. [30] and Hinrichs *et al*. [31] indicated that the best ES would be a microporous, compliant and biodegradable material. From the mechanical point of view, the PGA non-woven fabric is too rigid and should not be considered as an ideal material for ES. Although the PGA ES did not have a significant impact on eliminating the compliance mismatch, it strengthened the graft and prevented over expansion, and finally resulted in a much smaller cross-sectional area. Table 4 gives a quantitative comparison: the cross-sectional area of HM was around 15 times that of its host artery and 5 times more than the diameter of the original vein. On the other hand, that of HSM only increased by 50% compared with the original size. Even though the geometrical mismatch had not been eliminated by the ES totally, the blood flow pattern and the shear stress in the graft with ES are much more uniform, which is usually regarded as a crucial factor on the function of the EC and its denudation [32]. In this study we found that EC was preserved by the ES in comparison with the denudation of EC and exposure of subendothelial tissue in the grafts without protection (the pictures from scanning electronic microscope are not shown here). Without protection, the venous inner layer could be over stretched by about 3 times under arterial pressure, causing rupture of the joint between ECs and basilar membrane. Therefore in our opinion, over-expansion also plays an important role in the denudation of the EC.

**Table 4:** Cross section areas in load free state (*mm*^2^)

	**HA**	**HV**	**HSM**	**HM**
*A*_*non-load*_	1.28 ± 0.11	2.36 ± 0.64	3.87 ± 0.17	18.15 ± 7.91

All groups are much significantly different from each other (P < 0.01).

From the reported results, ESs have a quite similar protection effect, such as preventing hyperplasia in the inner layer, preserving EC and SMC, regulating the extracellular matrix, reducing the lipid deposition, stopping the granulocyte and macrophage invasion and the formation of foam cells, and finally improving the bypass patency significantly. However Es currently has not been applied clinically. This is mainly because of the following remaining questions: (1) how the mechanical property of the graft varies during the remodeling process when different kinds of ESs are used, (2) what is the long-term outcome, (3) what is the standard operation process of cuffing, and (4) what is the ideal material for ES. Further studies to address these issues would be very useful.

## Conclusion

Grafts both with and without ES displayed a large compliance and high expansibility in the low pressure range and were almost inextensible in the high pressure range, and such behavior was more prominent in those without ESs. The collagenous fiberized graft wall and the disappearance of elastic fiber were the key factors leading to this mechanical behavior. Although the PGA ES reduced the compliance mismatch between graft and host artery, the gap remained very large after 8 weeks. Therefore from the mechanical point of view, the PGA non-woven fabric is not an ideal material for ES. However, its effect is obvious in reducing the geometrical mismatch, which is important for the shear stress pattern in the graft.

## Abbreviations

EC Endothelial cell

ES External stent

HA: Non-touched artery from hypercholesterolemic animal

HD: Hypercholesterolemic diet

HDL: High density lipoprotein

HH: For the hypercholesterolemic animal, whose graft is without external stent and not for mechanical test

HM: For the hypercholesterolemic animal, whose graft is without external stent and for mechanical test

HSH: For the hypercholesterolemic animal, whose graft is with external stent and not for mechanical test

HSM: For the hypercholesterolemic animal, whose graft is with external stent and for mechanical test

HV: Non-touched vein from the hypercholesterolemic animal

LDL: Low density lipoprotein

NDG: Control group with normal diet

PGA:Polyglycolic acid

SMC: Smooth muscle cell

TC:Total cholesterol

TG: Triglyceride

## Competing interests

The author(s) declare that they have no competing interests.

## Authors' contributions

All authors have read and approved the final manuscript.

Zhongzhao Teng and Guangyu Ji have made substantial contributions to this study, including conception, design, acquisition of data, analysis and interpretation of data and drafting the manuscript.

Hongjun Chu took apart in surgical operations, mechanical testing, histological examination and acquisition of data.

Zhi-Yong Li revised the manuscript critically for important intellectual content.

Liangjian Zou and Zhiyun Xu also contributed to the conception and design, provided funding and valuable guidance for this research.

Shengdong Huang was involved in the surgical operations.
